# A liver health hui: hepatitis C knowledge and associated risk factors in New Zealand gang members and their families

**DOI:** 10.1098/rsos.172167

**Published:** 2018-08-29

**Authors:** Kristina Aluzaite, Jordan Tewhaiti-Smith, Margaret Fraser, Steve Johnson, Elizabeth Glen, Allison Beck, Barbara Smith, Jack Dummer, Michael Schultz

**Affiliations:** 1 Department of Medicine, University of Otago, PO Box 913, Dunedin 9054, New Zealand; 2 Gastroenterology Unit, Southern District Health Board, Dunedin Hospital, Dunedin, New Zealand; 3 Otago Hepatitis C Resource Centre, Dunedin, New Zealand; 4 Dunedin Intravenous Organization Needle Exchange, Dunedin, New Zealand

**Keywords:** hepatitis C, hepatitis B, New Zealand, gang population, liver health

## Abstract

Hepatitis C virus (HCV) and B virus (HBV) infections are highly prevalent, with a high percentage of undiagnosed cases. Knowledge of HCV and its modes of transmission are essential for disease prevention and management. We studied a high-risk New Zealand gang population on viral hepatitis prevalence, their level of knowledge and the liver health risk factors in a community setting. Participants completed demographic, risk factor and knowledge questionnaires in three health meetings in New Zealand. Participants' blood samples were tested for HBV, HCV, biochemical indicators of liver disease. Liver fibrosis levels were assessed using a Fibroscan^®^ device. We studied 52 adult Mongrel Mob members, affiliates and whānau (extended family) throughout New Zealand. We identified no HCV and two HBV cases, confirmed high-risk factor levels and poor associated knowledge, with a significant association between lack of knowledge and presence of specific risk factors. We successfully conducted a community-focused, high-risk, hard-to-reach gang population study, and found a link between lack of knowledge and risk factors for HCV infection. This study provided first-of-its-kind data on viral hepatitis in a gang population and demonstrated the need for educational screening programmes to aid early HCV detection, prevention and treatment.

## 1. Background

Infection with the hepatitis C virus (HCV) is a major cause of chronic liver disease and a major risk factor for complications such as liver cirrhosis and hepatocellular carcinoma [1–3]. It is estimated that 2% of the world's population is HCV-viraemic, not excluding New Zealand: a recent study found 4% HCV prevalence in 40–59-year-old people in Dunedin [4,5] and 1% (approx. 50 000 infected people) in the overall New Zealand population [6]. It is likely that the epidemiological data on HCV are incomplete due to asymptomatic cases and poor reporting [6]. Despite the fact that HCV infection is curable, it is estimated that 20–75% of infected people are not aware of their infection [6–10], resulting in 70–85% of them remaining chronically infected [11,12]. Early detection and treatment of HCV are instrumental in preventing transmission, liver damage and end-stage liver disease.

HCV infection risk factors include intravenous drug (IVD) use—an estimated 79.4% of people with an HCV infection have a history of recreational IVD use [13]—and percutaneous transmission via unsterile tattooing, cosmetic and body-piercing procedures [14]. As a result, prisoners, IVD users and gang members are generally considered high-risk groups [14]. A number of studies have been conducted describing the presence of HCV in prison populations, reporting a high prevalence with a considerable variation [15]: from 2.4% in Brazil [16] to 34.3% in California, USA [17], and 29% in Australia [18]; however, to our knowledge, there are no studies available on hepatitis C, its epidemiology and associated factors in other high-risk gang populations.

Gangs, in addition to being associated with high-risk activities, are also considered hard-to-reach populations and are often marginalized in societies. The Mongrel Mob is the largest gang in New Zealand with approximately 1510 members making up 38.1% of the total country's gang population (based on known adult gang members, July 2014) [19,20]. Owing to factors such as high incarceration rate, common IVD use and uncertified tattooing, their members and relatives are considered to be a high HCV risk group. Other than viral hepatitis, factors such as high alcohol consumption and obesity also contribute to poor liver health [21], and are probably highly prevalent in gang populations.

Furthermore, poor knowledge about hepatitis C transmission and treatment options is itself an important HCV risk factor [22]; low general level of education is also associated with higher infection rates [23]. Successful knowledge provision is often complicated in hard-to-reach marginalized groups, such as gang members and their families due to social stigma, which is identified as one of the key sources of barriers in hepatitis C management [24,25]. There is a widespread belief that these people are difficult to engage with, and few initiatives take place to fill the existing gaps. We had a unique opportunity to study a marginalized group, generating the first data of this kind.

A number of studies showed improvements to hepatitis-related knowledge, changes in behaviour and increased willingness to commence treatment and/or take preventative strategies after brief educational interventions [22,26]. Some studies reported high interest in free HCV screening programmes [26], where 99% of studied HCV-infected people supported free HCV testing in their communities. It was also found that a brief educational intervention led to significant improvements in participants' level of understanding of hepatitis C [26].

The goals of this study were to estimate HCV and HBV prevalence, and markers of general liver health in New Zealand gang members and their families, assess their level of knowledge associated with modes of infection and treatment, as well as presence of risk factors. We also simultaneously indirectly tested a new approach of accessing a hard-to-reach group through an educational community ‘health hui’—hui being the indigenous Māori word for social assembly or meeting.

## 2. Methods

### 2.1. Study design and participants

The study participants included Mongrel Mob Notorious chapter gang members and affiliates,^1^ and their family members due to anticipated shared risk factors for hepatitis B and C infections and chronic liver disease. The meetings took place in Dunedin, Lower Hutt (Wellington) and Turangi (Taupo), New Zealand.

Blood samples were taken by phlebotomists from Southern Community Laboratories (Dunedin, New Zealand). Participants were asked to complete demographic, HCV/HBV knowledge and risk factor questionnaires, provided a blood sample and had a Fibroscan^®^ examination. Data on participants' height and weight were collected for BMI calculations for the Turangi participants.

The information about the events was distributed via word-of-mouth and social media within the communities. There was no financial or other compensation, but the participants received an educational session about HCV modes of transmission and treatment options at the end of the meetings. Furthermore, any concerning results were sent back to participants, if requested.

### 2.2. Blood samples

Blood samples were taken and analysed by the Southern Community Laboratories’ (Dunedin, New Zealand) staff. The samples were tested for HBV surface (*Roche Cobas^®^*^2^ *Anti-HBs*) and core (*Roche Cobas^®^ Elecsys Anti-HBc II)* antibodies, HBV surface antigens (*Roche Cobas^®^ HBsAH II quant II*), HCV antibodies (*Roche Cobas^®^ Anti-HCV II*) and biochemical indicators of liver health: alanine transaminase (ALT) (*Roche Cobas^®^ ALT*) and γ-glutamyl transpeptidase (GGT) (*Roche Cobas^®^ GGT-2*). The following scores were indicative of elevated levels: ALT greater than 29 IU l^−1^ for men and greater than 22 IU l^−1^ for women [27]; GGT: greater than 51 U l^−1^ for men and greater than 33 U l^−1^ for women [28].

### 2.3. Liver fibrosis

A non-invasive Fibroscan^®^ [29] was used to assess the level of fibrosis in the liver. Fibrosis stage was reported using the METAVIR scoring system: F0 (less than 7.1 kPa): normal; greater than or equal to F1 (greater than or equal to 7.1 kPa): indicative of minimal fibrosis; greater than or equal to F2 (greater than or equal to 7.8 kPa): significant fibrosis; greater than F3 (greater than 8.0 kPa): severe fibrosis; and greater than F4 (greater than 11.5 kPa): cirrhosis/scarring [30].

### 2.4. Statistical analysis

Descriptive statistics on the study cohort demographics, HCV knowledge and risk factors were derived. A *χ*^2^ test for independence was used to determine if there was any association between tested knowledge levels and exhibited risk factors. Simple linear regression (for participants' age), two-sample *t*-test (for participants’ sex) and one-way ANOVA with Tukey HSD post hoc test (for participants' gang affiliation status and level of education) were used to explore association of demographic variables with knowledge questionnaire scores; two participants were not included in this analysis due to low questionnaire completion. No multiple correction was done in order to report all possible relevant associations for future studies. *p*-values at or below 0.05 were considered statistically significant. Missing data entries were omitted from descriptive statistic calculations for each factor. Data analysis was performed using the R statistical computing language [31].

## 3. Results

### 3.1. Study population

Fifty-three adult Mongrel Mob Notorious gang chapter members, affiliates and their whānau (extended family) were recruited for the study in three New Zealand cities—Dunedin, Wellington and Turangi (table 1). One participant was removed due to inadequate questionnaire completion. Of the total studied population (*n* = 52): 34.6% (*n* = 18) were gang members, 55.8% (*n* = 29) were whānau (extended family) and 9.6% (*n* = 5) gang affiliates.

**Table 1.** Characteristics of the study population.

participant characteristics	% (*n*)
type of participant	
gang member	34.6% (*n* = 18)
affiliate	9.6% (*n* = 5)
extended family/Whānau	55.8% (*n* = 29)
sex, male	51.9% (*n* = 27)
age (years), median (IQR)	36.0 (27.5–43.0)
study location	
Dunedin	25% (*n* = 13)
Wellington	36.5% (*n* = 19)
Turangi	38.5% (*n* = 20)
ethnicity	
Māori	75% (*n* = 39)
education	
below secondary	19.6% (*n* = 10)
secondary	47.1% (*n* = 24)
tertiary	11.8% (*n* = 6)
undergraduate	5.9% (*n* = 3)
graduate	15.7% (*n* = 8)
regular GP^a^ visits	
weekly–monthly	17.6% (*n* = 9)
6 monthly	37.3% (*n* = 19)
yearly	9.8% (*n* = 5)
5 yearly/rarely	35.3% (*n* = 18)
perceived BMI	
underweight	5.9% (*n* = 3)
normal weight	35.3% (*n* = 18)
overweight	54.9% (*n* = 28)
excessively overweight	3.9% (*n* = 2)
BMI (Turangi cohort)	(*n* = 20)
overall median (IQR)	32.9 (28.7–36.1)
normal (BMI = 24)	5% (*n* = 1)
overweight (25.0–29.9)	25% (*n* = 5)
obesity class I (30.0–34.9)	40% (*n* = 8)
obesity class II and III (greater than 35.0)	30% (*n* = 6)

^a^General practitioner.

About 51.9% (*n* = 27) of the participants were male and 75% (*n* = 39) considered themselves of Māori heritage. The median age of the participants was 36 years (IQR = 27.5–43.0 years), and 33.3% (*n* = 17) had higher than secondary education.

### 3.2. Prevalence of hepatitis C virus

No cases of HCV infection were identified in the study population. Two participants were found to be hepatitis B virus carriers.

### 3.3. Liver health

About 18.9% (*n* = 10) of study participants had evidence of liver fibrosis: two, four and four people scored F2 (significant fibrosis), F3 (severe fibrosis) and F4 (cirrhosis/scarring), respectively. Sixty per cent (*n* = 6) and 50% (*n* = 5) of these people had elevated ALT and GGT, respectively. The median liver stiffness measurements were comparable among the BMI categories for the Turangi participants (*n* = 20) with 4.7, 4.5 and 4.4 kPa Fibroscan readings for BMI 25–29.9, 30–34.9 and greater than or equal to 35, respectively (electronic supplementary material, appendix, table S1).

About 39.2 and 33.3% of participants in the overall study population had elevated ALT and GGT, respectively; these markers were not correlated to BMIs in the Turangi population.

### 3.4. Hepatitis C risk factors

**Figure 1.**
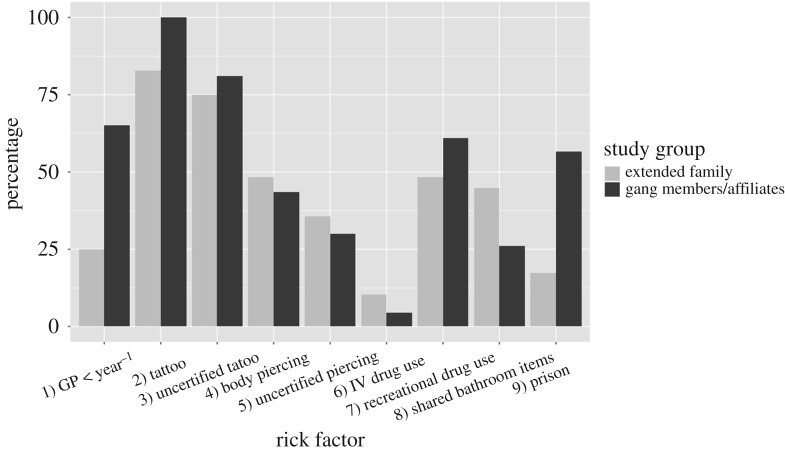
Risk factor prevalence comparison between the combined gang member and affiliate, and whānau (extended family) groups.

As expected, the Mongrel Mob Notorious study population proved to be at high risk for HCV infections. About 7.7% of the study population currently are, or have previously been injecting drug users, and 53.8% have used recreational drugs. About 34.6% of the study group have been in prison. Finally, 90.4% of the study participants had tattoos and 46.2% had piercings, 73.8 and 30.4% of which, respectively, were done via uncertified outlets. The prevalence of risk factors was similar between the gang member/affiliate and the extended family (figure 1) groups, except for incarceration rates (54.2% (*n* = 13) of gang members and affiliates, and 17.2% (*n* = 5) of whānau).

Data on participants’ weights were only available for the Turangi group (38.5% (*n* = 20) of the study population), where the median (IQR) BMI was 32.9 (28.7–36.1). Seventy per cent (*n* = 14) of the participants were obese (BMI ≥ 30.0) (table 1). When comparing these data with participants' perception of their body mass, it was found that 78.9% of participants underestimated their BMI category.

In the full cohort (*n* = 52), 58.8% of the participants perceived themselves to be overweight, which is probably an underestimate of their true BMI, considering the Turangi observations outlined above. About 35.3% visited their GP less than once a year. Only 21.2% of the overall study population and 26.3% (*n* = 10) of those under 42 (eligible for a free vaccination programme for those under 18 introduced in 1991 [32]) thought they had received an HBV vaccination, while the HBV serological test results indicated HBV immunity in 55.6% (*n* = 15/27) of those eligible for a free HBV vaccination. Furthermore, only 30.0% (*n* = 3/10) of the participants who thought they had been vaccinated, had serological markers of HBV immunity.

### 3.5. Alcohol consumption

**Figure 2.**
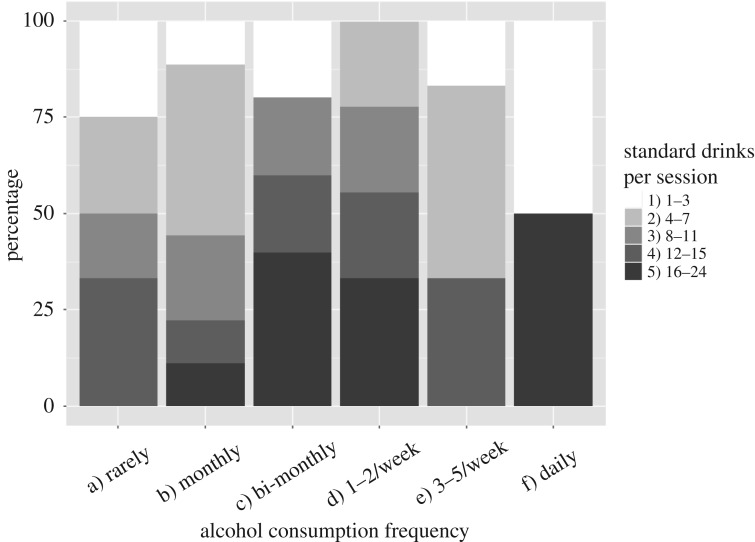
Alcohol consumption patterns in the study population—consumption frequency and quantity of standard drinks per session.

About 38.5% (*n* = 20) of the study participants consumed alcohol at least once a week, and 56.8% (*n* = 25) consumed at least eight standard drinks per drinking session (figure 2). About 73.1% (*n* = 38) of the study participants consumed more than the recommended alcohol amount [33].

The study population overall was highly diverse in its drinking patterns (figure 2).

### 3.6. Knowledge on hepatitis C

On average, the study population scored 45.3% (median 43.3%; IQR = 33.3–56.7) in the viral hepatitis knowledge questionnaire (table 2). The participants of the population scored poorly in the question about HCV transmission; only 51 and 41% answered correctly that hepatitis can be transmitted through unsterile medical and cosmetic procedures, and contact sport/fighting, respectively. The majority of the study participants correctly identified that hepatitis B and C can be treated (60 and 57%); however, only 20% knew that the treatment is funded in New Zealand. Surprisingly, 88% of the participants severely overestimated the prevalence of hepatitis in New Zealand, with 39% of the participants stating that the infections are as common as 50%. Only 43–63% of the study population knew that liver cirrhosis and scarring, liver cancer and liver failure were possible HCV infection outcomes; 18% of the participants thought there were no long-term disease-related complications. Finally, only 29–43% of people correctly identified HCV infection-related symptoms.

**Table 2.** Hepatitis C virus-associated knowledge questionnaire response summary.

question	% correct answers (*n* = 52)
1. How hepatitis B and C are spread/transmitted?	
sharing a toothbrush or razor with an infected person	65% (*n* = 33)
sharing needles, spoons, filters (IV drug utensils)	86% (*n* = 44)
through sexual intercourse	63% (*n* = 32)
from mother to baby at birth	55% (*n* = 28)
unsterile medical/dental/cosmetic procedures (tattoos/piercings)	51% (*n* = 26)
contact sport/fighting	41% (*n* = 21)
occupational hazards (exposure to blood in the work place)	59% (*n* = 30)
2. Can a hepatitis infection be treated?	
hepatitis B	60% (*n* = 30)
hepatitis C	57% (*n* = 25)
3. Is treatment for hepatitis funded in New Zealand?	20% (*n* = 9)
4. Who would you approach to get treatment?	
GP	80% (*n* = 41)
gastroenterologist	4% (*n* = 2)
infectious disease specialist	20% (*n* = 10)
5. How common do you think viral hepatitis is in New Zealand?	50–39% (*n* = 20)
	20–31% (*n* = 15)
	10–16% (*n* = 8)
	5–2% (*n* = 1)
	1–2% (*n* = 1)
6. Can you be vaccinated against:	
hepatitis B (‘yes’; ‘don't know’)	39% (*n* = 20); 53% (*n* = 27)
hepatitis C (‘no’; ‘don't know’)	10% (*n* = 5); 58% (*n* = 28)
7. What are the possible long-term problems associated with hepatitis?	
liver cancer	47% (*n* = 23)
liver cirrhosis/scarring	43% (*n* = 21)
liver failure	63% (*n* = 31)
no long-term problems	18% (*n* = 9)
8. Symptoms of hepatitis B/C infection can include	29–43% correctly identified different symptoms
9. How long does it take for symptoms of a chronic infection to be noticed?	never—11% (*n* = 5)
	days—21% (*n* = 10)
	weeks—15% (*n* = 7)
	months—30% (*n* = 14)
	years—28% (*n* = 13)

**Figure 3.**
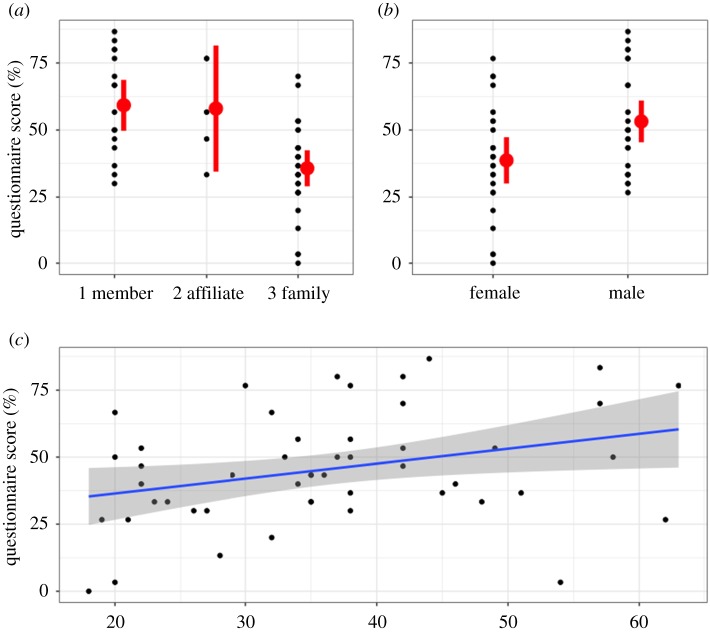
Differences in the overall hepatitis knowledge questionnaire scores based on (*a*) gang affiliation; (*b*) gender; (*c*) participants' age; presented with 95% confidence intervals.

Gang affiliation status was significantly associated with the hepatitis knowledge questionnaire scores (*p* *=* 0.014); whānau (extended family) scored lower than the gang members (*p* *=* 0.00027) and affiliates (*p* *=* 0.035) (figure 3*a*). Female gender (*p* *=* 0.013) and younger age (*p* *=* 0.026) were also significantly associated with worse questionnaire results (figure 3*b,c*). There was no association between level of education and viral hepatitis knowledge (*p* *=* 0.11).

While we could not compare levels of knowledge between those infected with HCV and not, we identified a significant association between lack of HCV-related knowledge and risky behaviour. There was evidence that being unaware of potential HCV spread through shared use of ‘bathroom items’, such as a toothbrush or a razor, was associated with this risk factor (*p* *=* 0.033).

In addition to collecting highly valuable epidemiological data, we were able to create a platform for health education outreach and engagement; we received a lot of interest from the community and were able to reach the target gang and affiliate population, as well as their families and relatives.

## 4. Discussion

This study is the first of its kind to engage with a hard-to-reach gang population to study prevalence, risk factors and knowledge about viral hepatitis. We did not identify any cases of hepatitis C in the study population but two hepatitis B carriers were found. Overall, our study confirmed the high-risk status of this population and demonstrated a low level of knowledge regarding transmission, symptoms, risk factors and available treatment options for viral hepatitis.

We were unable to confirm the prevalence of hepatitis C in this population, which was thought to be higher than the national average [6]; in a previous study, we found up to 4.0% of Dunedin's middle-aged general population to be positive for HCV [5]. This is an unexpected result, and could be explained by a small population size (given an estimated 1.5–4% prevalence of anti-HCV in New Zealand adults, 0.78–2.08 persons would be expected to be positive in the study population of 52) and the self-selecting nature of the study design. While 56% of the tested population where whānau, which was thought to contribute to under-estimating the prevalence of HCV in the population of interest, the prevalence of HCV risk factors was similar across the different gang affiliation statuses.

There were several concerning facts about the general liver health in the study population. We identified three times higher rates of elevated ALT and GGT levels compared to the general New Zealand population (39.2 and 33.3% versus 13.1 and 13.7%, respectively) [21]. Moreover, one-fifth of the study population demonstrated significant to severe levels of fibrosis and cirrhosis. This is exceptionally worrying, given the relatively young age of the cohort (median = 36 years). We also found exceptionally high levels of alcohol consumption both in frequency and quantity, which was at least two times higher than the already high national average—it is estimated that 17% of New Zealand population is involved in harmful or hazardous drinking [34]. Furthermore, we found more than two times higher obesity rates (70% of the Turangi population was obese) than the national statistic (32% of adults in New Zealand are obese [35]) that probably have contributed to poor liver health. When comparing perceived and actual BMI, 78.9% of the Turangi participants' group underestimated their BMI category. The findings are probably generalizable to the overall study population, and suggest a very poor understanding of the healthy weight range and the need for further education. Given that 35.3% of the population visited their GP less than once every 5 years, these are highly concerning findings.

As expected, the studied Mongrel Mob Notorious chapter gang population was a high hepatitis C risk group with a very high incarceration rate (34.6%) (in contrast with 1.55% national New Zealand rate [36]), high IVD use (7.7%) and a very common use of uncertified tattoo and piercing outlets (73.8 and 30.4%, respectively). What is important is that in combination with high-risk factors’ presence, the group exhibited poor hepatitis C-related knowledge. Approximately half of the population failed to correctly identify HCV modes of transmission and potential symptoms of an acquired infection. Moreover, half of the study population was not aware and/or underestimated the severity of long-term health problems associated with HCV that highlights lack of understanding of the implications of the disease and suggests an existing perception that HCV is a benign infection [37]. Finally, the study participants were not aware of their HBV vaccination status, as suggested by the inconsistencies between the self-reported vaccination status and the serum markers of HBV immunity.

On average, the Mongrel Mob Notorious population scored 43.3% in the questionnaire, with the 75th percentile (score 56.7%) being lower than the average knowledge score of 59.4% in the Dunedin general population [5]. Owing to the nature of the study, it is still likely that it overestimates level of knowledge in NZ gang population. Unlike in the general Dunedin population, there was no significant difference between the general level of education of the study participants and their scores; however, we identified females, younger participants and the extended family to be significantly less knowledgeable about the topic. Given that this marginalized group had lower rate of post-secondary education than the national New Zealand average (33.3 versus 39% [38]), incorporating viral hepatitis prevention education into secondary school curriculum could help resolve the problem.

We found a significant association between HCV-related level of knowledge and presence of risky behaviours; study participants who did not know certain modes of HCV transmission, were more likely to exhibit these risk factors. That shows that HCV education plays an important role in infection prevention. A number of studies have shown efficacy of educational interventions to raise hepatitis C awareness [26]. Better disease prevention, diagnosis and treatment will contribute to decrease rates of liver cirrhosis and hepatocellular carcinoma [39].

To our knowledge, to date there are no data available of hepatitis C prevalence and associated factors and knowledge in gang populations. Our study provides a unique insight into a very important high-risk marginalized hard-to-reach population.

This study is an example of an epidemiological investigation combined with an outreach initiative. We were able to not only study a high-risk population, but have a unique chance to engage an entire community and provide knowledge that is likely to disseminate outside the immediate study group. We hope this study will serve as an example for a number of similar initiatives in the future.

## Supplementary Material

Appendix

## Supplementary Material

Complete_data_healthhui.xlsx

## Footnotes

1 Gang affiliate is defined as someone associated with the gang, but not yet accepted as an official gang member and not a biological family member.

2 Roche Diagnostics GmbH, Sandhofer Strasse 116, 68305 Mannheim, Germany.

## Data accessibility

The study data file is available in the electronic supplementary material. We removed the participants' location and employment information to protect their privacy.
