# Supplementary material for: A liver health hui: hepatitis C knowledge and associated risk factors in New Zealand gang members and their families
Source: R Soc Open Sci. 2018 Aug 29;5(8):172167. doi: 10.1098/rsos.172167 (PMC6124044; doi:10.1098/rsos.172167)
Supplement: Appendix [file rsos172167supp1.docx]

*Table 1: Fibroscan Liver stiffness estimates in the BMI groups (Turangi population, n=20)*

| BMI group (kg/m^2^) | Median (IQR) liver stiffness, kPa |
| --- | --- |
| Normal (<25) (n=1) | 4.3 (NA) |
| Overweight (25-29.9) (n=5) | 4.7 (4.4-4.8) |
| Obesity class I (30-34.9) (n=8) | 4.5 (4.0-5.9) |
| Obesity class II and III (≥35) (n=6) | 4.4 (4.1-11.2) |
